# Parametric Acoustic Array and Its Application in Underwater Acoustic Engineering

**DOI:** 10.3390/s20072148

**Published:** 2020-04-10

**Authors:** Hanyun Zhou, S.H. Huang, Wei Li

**Affiliations:** 1 Ocean College, Zhejiang University, Zhoushan 316000, China; 11634017@zju.edu.cn; 2 College of Electrical Engineering, Zhejiang University, Hangzhou 310027, China; 11610059@zju.edu.cn

**Keywords:** parametric acoustic array, nonlinear acoustics, signal processing techniques, underwater acoustic engineering

## Abstract

As a sound transmitting device based on the nonlinear acoustic theory, parametric acoustic array (PAA) is able to generate high directivity and low frequency broadband signals with a small aperture transducer. Due to its predominant technical advantages, PAA has been widely used in a variety of application scenarios of underwater acoustic engineering, such as sub-bottom profile measurement, underwater acoustic communication, and detection of buried targets. In this review paper, we examine some of the important advances in the PAA since it was first proposed by Westervelt in 1963. These advances include theoretical modelling for the PAA, signal processing methods, design considerations and implementation issues, and applications of the PAA in underwater acoustic engineering. Moreover, we highlight some technical challenges which impede further development of the PAA, and correspondingly give a glimpse on its possible extension in the future. This article provides a comprehensive overview of some important works of the PAA and serves as a quick tutorial reference to readers who are interested to further explore and extend this technology, and bring this technology to other application areas.

## 1. Introduction

Due to the motion nonlinearity and medium nonlinearity, an end-fire array of virtual sources of the difference frequency can be obtained when two intense waves with slightly different frequencies propagate in the same medium. The virtual end-fire array is referred to as parametric acoustic array (PAA). PAA was first proposed and analyzed in the early 1960s by Westervelt [1], who changed the previous conception that low-frequency sound waves could not form sharp directivity. Since then, there has been great interest in studying and developing the theory of the PAA. After decades of research, the PAA has been moved from theory and experimentation to implementation and application. The nonlinear acoustical theory and the theoretical models of the PAA have been developed and investigated intensively. Dual-frequency parametric array and broadband parametric array are two main models of the PAA, with the latter more widely used in practice because of its broad bandwidth advantage. Despite this advantage, the secondary wave produced by broadband parametric array has high levels of distortion. Several signal processing techniques therefore have been developed to generate expected signals and reduce distortion. Besides, to achieve the best performance, a compromise between various factors including primary frequencies, secondary frequency and transducer array size should be made when designing the PAA. Therefore, according to the specific application scenarios, some design considerations and guidelines also have been studied. Due to its prominent advantages, such as low frequencies with high directivity, small and constant beam-width, side-lobe free beam patterns, and broadband capability at low frequencies, PAA has been widely applied in the fields of underwater acoustic engineering, such as sub-bottom profile measurement, underwater acoustic communication, detection of buried targets, etc. However, some technical bottlenecks which impede the further development and application of the PAA remain to be settled. They are concerned with transmitting transducer performance, conversion efficiency, power consumption, signal processing and modulation, and detection efficiency. This review paper provides a tutorial overview of the foundation work in the PAA and its application in underwater acoustic engineering. Further, we give a glimpse on the recent developments in the PAA and the emphases for its future work.

This paper is organized as follows. The fundamental principles of the PAA are introduced in Section 2, including a brief summary of nonlinear acoustic theory related to the PAA and theoretical models of the PAA. This is followed by the analysis and comparison of several signal processing and modulation techniques for the PAA in Section 3. Next, Section 4 outlines some design considerations and implementation issues on developing the PAA system. Several successful applications of the PAA in underwater acoustic engineering are then presented in Section 5. Main technical challenges and related researches are highlighted in Section 6. Finally, Section 7 concludes this paper with some directions for future research.

## 2. Fundamental Principles of the PAA

### 2.1. Nonlinear Acoustic Theory

**Figure 1:**
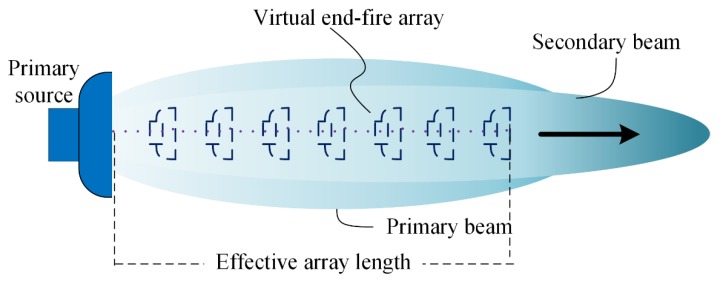
Generation of the secondary beam through the PAA.

Linear acoustics ignore the effects of motion nonlinearity and medium nonlinearity, leading to a linear acoustic law—when two acoustic waves of different frequencies propagate in the same medium, they do not interact with each other, and the total sound field is a linear superposition of the two waves. From the perspective of nonlinear acoustics, however, when two waves travel in the same direction, each wave propagates in the medium with another wave’s disturbance resulting in scattering due to the inhomogeneity of the medium. This phenomenon is called the scattering of sound by sound [2]. As two sound waves of different frequencies with finite amplitude interact with each other in a medium, distorted waves with the sum, the difference and harmonic components can be generated due to the nonlinearity of the medium. However, only the difference frequency component can travel a long distance because sound absorption is increased with frequency generally. Therefore, the characteristic of difference frequency sound field is the main concern of the PAA. The secondary source column of the difference frequency is virtually created in the primary beam and is distributed along a narrow beam, which is similar to an end-fire array (as shown in Figure 1). According to the directivity characteristics of the end-fire array, the obtained difference frequency beam has narrow beam-width and high directivity.

The nonlinear sound field is described above from the perspective of mutual scattering of sound waves. Another important theory of nonlinear acoustics is finite amplitude effect. The instantaneous sound speed depends not only on the medium but also on the sound pressure or particle velocity [3]. The velocity is the highest at the crest and the lowest at the trough. As the propagation goes on, the waveform becomes distorted gradually, and the sine wave turns into saw-tooth wave, even shock wave (i.e., appearing harmonics).

The two primary frequency waves produce scattered sound during the propagation, and the forward scattering part (i.e., the part with the same propagation direction of the primary frequency wave) is superimposed on the previously generated sound in the same direction to gradually strengthen the sound field. Hence, the sound field of the PAA is an accumulative field, with the total energy of the difference frequency wave increases as the propagation distance increases. Even after deducting the absorption effect of the difference frequency wave, the source level measured in the far field is still higher than that measured in the near field. Therefore, to accurately measure the source level of the parametric array, the measuring point must be far enough away from the source.

### 2.2. Theoretical Model of the PAA

#### 2.2.1. Collimated Plane Wave Model

The theory of nonlinear interaction of sound waves was originally studied by Westervelt [1], who derived non-homogeneous wave equation based on Lighthill theory:

(1) $$p_{d}=\frac{\omega_{d}^{2}p_{1}p_{2}\beta S_{0}}{8\pi \rho_{0}c_{0}^{4}r}\frac{1}{\sqrt{\alpha^{2}+k_{d}^{2}\sin^{4}\frac{\theta }{2}}}$$

where $p_{d}$ is the difference frequency pressure produced by the nonlinear interaction of a pair of superimposed collimated beams, $\omega_{d}$ and $k_{d}$ are the angular frequency and wave number of the difference frequency signal, $p_{1}$ and $p_{2}$ are pressure amplitudes of two primary waves, β is the nonlinearity coefficient of the medium (for sea water, β≈3.6; for fresh water, β≈3.1), $S_{0}$ is the cross-sectional area of the sound beam, $\rho_{0}$ and $c_{0}$ denote the density of the medium and the sound velocity in the medium, r is the acoustic wave propagation distance, θ is the direction angle, $\alpha =\alpha_{1}+\alpha_{2}$, where $\alpha_{1}$ and $\alpha_{2}$ are the absorption coefficients of the two primary waves, and 1/α is usually called the efficient length of the virtual end-fire array.

There are some conclusions drawn from the Equation (1):

1. The difference frequency signal pressure $p_{d}$ is proportional to the product of the pressure of two primary waves. Normally, $p_{1}=p_{2}=p_{0}$ and one derives $p_{d}\propto p_{0}^{2}$.That is, if the primary source level drops by 3 dB, the secondary source level will drop by 6 dB.
2. $p_{d}$ is proportional to the square of the frequency of the difference frequency signal. In other words, when the secondary frequency doubles, the secondary source level will increase by 12 dB.
3. The half power beam-width (i.e., 3 dB beam-width) of the difference frequency signal can be approximately calculated as $2\theta_{-3\mathrm{dB}}\approx 4{\left(\frac{\alpha }{k_{d}}\right)}^{\frac{1}{2}}$. Since $\theta_{-3\mathrm{dB}}\propto {\left(\frac{1}{f_{d}}\right)}^{\frac{1}{2}}$, the beam-width of the difference frequency wave is relatively insensitive to the difference frequency, although the beam-width increases with the decrease of the difference frequency.

#### 2.2.2. Spreading Effect and Acoustic Saturation

Westervelt deduced Equation (1) on the assumption that the primary waves were collimated beams. It is assumed that the beam is so narrow that the volume distribution of the sources can be represented by the line distribution located along the axis of the primary beams. This assumption is valid within the Rayleigh distance only (i.e., $R_{F}=S/\lambda$, where S is the transducer surface area and λ is the wavelength of the primary wave). Beyond the Rayleigh distance, considering the spherical spreading effects, primary waves cannot be approximated by collimated plane waves. Berktay and Leathy took the spreading dissipation and directivity of the two primary waves into consideration and gave another expression [4]:

(2) $$p_{d}=\frac{\omega_{d}p_{1}p_{2}\beta }{4\pi \rho_{0}c_{0}^{4}r}\exp((-\alpha_{d}+jk_{d})R)\times \int_{-\frac{\pi }{2}}^{\frac{\pi }{2}}\int_{-\frac{\pi }{2}}^{\frac{\pi }{2}}\frac{D_{1}(\gamma ,\varphi )D_{2}(\gamma ,\varphi )\cos\gamma }{A+jk_{d}(1-u)}d\gamma d\varphi ,$$

where $A=\frac{1}{2}(\alpha_{1}+\alpha_{2})-\alpha_{d}$, and $\alpha_{d}$ is the absorption coefficient of the difference frequency signal, u=cosγcosθcos(φ−η)+sinγsinθ, θ,η are the direction angles of the field point, γ,φ are the azimuth angles of the source point, $D_{1},D_{2}$ are the directivity functions of the two primary waves, the other variables refer to the same meaning as those in Equation (1). Therefore, Westervelt’s approach to the calculation of non-linear effects in acoustic propagation is extended to the cases where the primary beams are spreading cylindrically or spherically.

In addition to the spread effect, acoustic saturation of the primary wave should also be taken into account when analyzing the sound field of the PAA. As we all know, the higher the primary source level, the higher the source level of the generated difference frequency signal. To increase the amplitude of the secondary wave, it is reasonable to increase the transmitted power of the primary signal. However, due to the finite amplitude effect, the amplitude of the secondary wave cannot increase indefinitely. When the propagation distance of the primary wave exceeds a certain value, the sine wave turns into the saw-tooth wave, and some of the energy in the primary wave is converted to harmonics, called “excess attenuation” [5]. The propagation distance from where the waveforms start to become distorted is called the “shock distance” [6]:

(3) $$R_{s}=\frac{1}{\beta kM},$$

where k is the original frequency wave number, and M is the acoustic Mach number defined by $M=v/c_{0}$ (i.e., the ratio of the vibration velocity of the medium particle near the transducer to the sound velocity). The saturation of the primary beam serves as the limiting mechanism that prevents the amplitude of the difference frequency signal from increasing indefinitely with the increase of the transmitted power.

#### 2.2.3. Summary of the Parametric Array Model

To summarize, there are three basic distances which will control the behavior of the PAA. They are efficient length of the PAA (or the absorption range), i.e., $R_{A}=1/\alpha$, the Rayleigh distance $R_{F}=S/\lambda$ and the shock distance $R_{s}=1/\beta kM$. Depending on the values of these three distances, different mathematical models have to be used to predict correctly the source levels of the secondary wave [6]:

- Absorption limited: $R_{s}>R_{F}>R_{A}$, in this case, both spreading and excess attenuation are ignored and Westervelt’s model can be used.
- Spreading limited: $R_{s}>R_{A}>R_{F}$, this model has been initiated by Berktay and Leahy and takes into account the effects of spreading, but not those of excess attenuation.
- Shock-wave limited: $R_{F}>R_{A}>R_{s}$, Moffett and Mellen gave a detailed analysis of this model [7].

To accurately evaluate parametrically generated sound fields, both spreading and excess attenuation should be taken into account. However, it is a challenge to adaptively use different models to accommodate with their corresponding conditions. The most useful model equation, to solve this challenge, is the Khokhlov–Zabolotskaya–Kuznetsov (KZK) parabolic wave equation, which combines nonlinearity, dissipation, and diffraction of a directive sound beam in the same order of magnitudes [8,9]. The KZK equation is described as:

(4) $$\frac{\partial^{2}p}{\partial \tau \partial z}=\frac{c_{0}}{2}\nabla_{⊥}^{2}p+\frac{\delta }{2c_{0}^{3}}\frac{\partial^{3}p}{\partial \tau^{3}}+\frac{\beta }{2\rho_{0}c_{0}^{3}}\frac{\partial^{2}p^{2}}{\partial \tau^{2}},$$

where p is the acoustic pressure, δ is the sound diffusivity related to sound absorption, β, $\rho_{0}$,$c_{0}$ has the same meaning as those in Equation (1), $\tau =t-r/c_{0}$ is the retarded time, z is the coordinate along the axis of the beam and $\nabla_{⊥}^{2}=\partial^{2}/\partial^{2}x+\partial^{2}/\partial^{2}y$ represents a Laplacian that operates in the x–y plane perpendicular to the axis of the beam (z axis). The KZK equation is widely used in the calculation and analysis of the secondary sound field [10,11,12]. However, it is difficult to solve the KZK equation analytically. Thereby, numerical methods are developed to obtain the solution [13,14,15]. On the other hand, the use of numerical techniques such as finite difference methods that solve the nonlinear wave equation usually leads to the long computation times, which prevents their use in real-time simulation as required in some sensor applications. To improve the computational efficiency of the sound field calculations, expansion techniques based on the idea that sound beams can be expressed as a series of base functions has been proposed. Using a series of base functions, such as Gaussian–Laguerre [16] and Gaussian [17,18,19,20] base functions, the sound field expressions can be found for the primary and secondary fields. Then, simple and computationally efficient solutions can be obtained.

In addition to the above three common theoretical models of the parametric array sound fields, truncated parametric array model is also important in underwater acoustic engineering [21,22]. When parametric arrays are used for sub-bottom profiling or buried objects detection in shallow water, the array length of the PAA is often restricted due to the existence of the subsea interface, where the characteristics of the medium have an abrupt change. This creates a truncated parametric array, which generates a difference frequency signal with increased beam width and decreased sound source level [23].

#### 2.2.4. Broadband Parametric Array

When the primary waves are two single frequency waves, the difference frequency signal is a sine wave with only one frequency component. This is called the dual-frequency parametric array. If the primary signals are broadband, an infinite number of frequency components interact to generate a broadband difference frequency signal, forming a broadband parametric array. In 1965, Berktay proposed the “Berktay far-field solution”, extending the theory of parametric array to the field of broadband signals. The Berktay far-field solution assumed the original wave of the PAA is a plane wave:

(5) $$p_{i}(t)=p_{0}E(t)\cos(\omega_{0}t),$$

where $p_{0}$ is the original frequency wave amplitude, E(t) is a function of the envelope, $\omega_{0}$ is the angular frequency of the original frequency wave.

Assume:

(6) $$p_{i}(r,t)=p_{0}e^{-\alpha_{0}r}E(t-r/c_{0})\cos(\omega_{0}t-kr),$$

where $\alpha_{0}$ is the absorption coefficient of the original frequency wave. By applying Equation (6) to the solution of the nonlinear equations of motion, further derivation can be obtained with respect to the difference frequency wave sound pressure [4]:

(7) $$p(t)=\frac{\beta p_{0}^{2}S_{0}}{16\pi \rho_{0}c_{0}^{4}r\alpha_{0}}\frac{d^{2}}{dt^{2}}E^{2}(\tau ),$$

Equation (7) is the Berktay far-field solution, which states that the difference frequency signal pressure is proportional to the second time-derivative of the square of the envelope of the amplitude-modulated carrier. That is, the nonlinear effect of the sound field can demodulate the envelope of the carrier, which is generally called the nonlinear self-demodulation model.

The self-demodulation model of the PAA has been widely recognized for decades. In addition, bandwidth is important both for communication and detection. Taking advantage of these two points, the broadband parametric array is more common than dual-frequency parametric array in practical engineering applications. However, the demodulated secondary wave predicted by Berktay’s far-field solution has high levels of distortion. An analysis of linear frequency modulated (LFM) pulse demodulated by the PAA is given here to indicate the severe distortion due to the parametric nonlinear effects. LFM pulse can be expressed as

(8) $$s(t)=A_{m}\times \mathrm{rect}(\frac{t}{T})\times \cos(2\pi (f_{0}t+\frac{k_{T}t^{2}}{2})),$$

where $A_{m}$ is the amplitude, $f_{0}$ is the starting frequency, $k_{T}$ is the tuning frequency and $\mathrm{rect}(\frac{t}{T})$ is a windowing signal whose value is equal to 1 when 0≤t≤T, and equal to zero at other times. According to Equation (7), the self-demodulated signal of LFM can be calculated as

(9) $$p_{d}=\frac{\beta p_{0}^{2}S_{0}A_{m}{}^{2}}{16\pi \rho_{0}c_{0}^{4}r\alpha_{0}}\times [-4k_{T}\pi \sin(4\pi (f_{0}t+\frac{k_{T}t^{2}}{2})-16\pi^{2}{\left(f_{0}+k_{T}t\right)}^{2}\cos(4\pi (f_{0}t+\frac{k_{T}t^{2}}{2}))].$$

**Figure 2:**
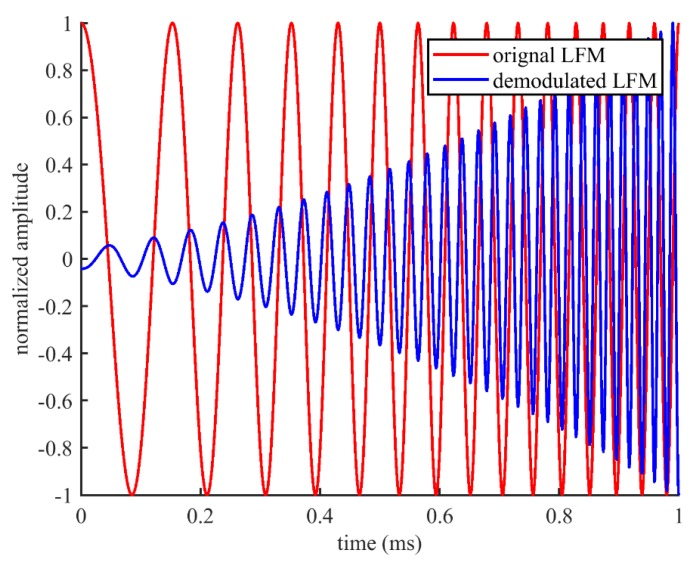
Comparison of the original linear frequency modulated (LFM) and self-demodulated LFM in time domain.

**Figure 3:**
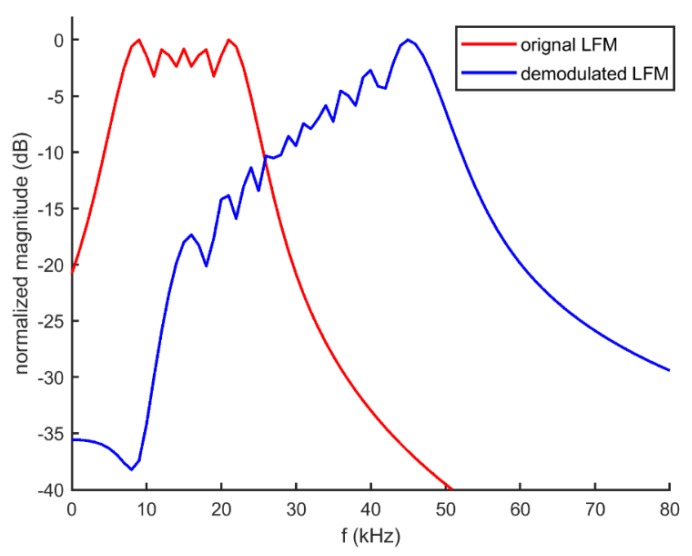
Comparison of the original LFM and self-demodulated LFM in frequency domain.

Figure 2 and Figure 3 show an example of the comparison between the original LFM and self-demodulated LFM, where the frequency range of the original LFM is 5–25 kHz and the time duration is T = 1 ms.

As can be seen, the self-demodulated LFM is strongly changed in comparison with the original LFM, and the amplitude of the high-frequency component is higher than that of the low-frequency component, which basically accords with the growth trend of 12 dB per octave. Moreover, the bandwidth of the secondary wave is approximately doubled relative to its original bandwidth. To reduce distortion and improve signal quality, signal processing and modulation techniques are required, which will be discussed in the following section.

#### 2.2.5. Recent Theoretical Development in the PAA

The analysis work published to date has dealt almost exclusively with the second-order nonlinear interactions of primary waves. Recently, it has been shown that third-order interactions derived from cascaded second-order interactions result in narrower sound beams. The improvement in beam pattern obtained by the higher-order nonlinear interactions has recently gained attention in a variety of applications, such as landmine detection [24,25], biomedical imaging [26,27,28], and nondestructive evaluation [29,30]. Besides, the third-order intermodulation (IM3) frequency components at $f_{L}=2f_{2}-f_{1}$ and $f_{U}=2f_{1}-f_{2}$ for primary frequencies $f_{1}$ and $f_{2}$($f_{1}>f_{2}$) are closely spaced in frequency to the primary waves, and thus the same transducer may be used to transmit the primary signals and receive the IM3 signals. This is in contrast to the secondary sum and difference components which are not close in frequency to the primary waves and would require a different transducer to be efficiently received. Therefore, more and more interest has been given to third-order and higher-order nonlinear interactions, leading to the development of models that account for higher-order interactions. A theoretical development of the third-order nonlinear scattering of sound from two noncollinear ultrasonic beams was presented by Garner and Steer [31], and they also carried out far-field and nearfield measurements to validate their theory. A more recent work was conducted by Johnson and Steer [32], who developed a computationally efficient model for third-order scattered sound fields using the series expansion of a set of Gaussian base functions. In summary the nonlinear interaction has been extended to include third-order and higher-order effects, and there is no doubt that the theoretical development in the PAA will further promote its wider applications in various fields.

## 3. Signal Processing and Modulation Techniques

**Figure 4:**
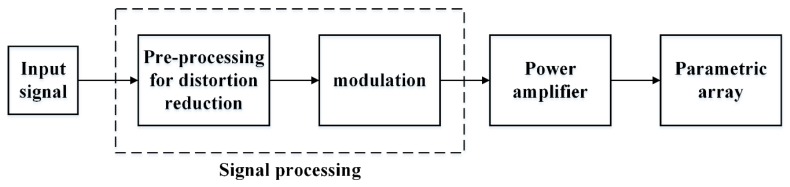
Block diagram of the PAA system.

Figure 4 shows the configuration of the PAA system. Signal processing module is an important part of the system, including two main functions, namely pre-processing and modulation. As mentioned in Section 2, the demodulated secondary waveform is distorted due to the nonlinear effects, so pre-processing methods are necessary and important for generating the expected signals, such as LFM signal, digital modulated signals, etc. Pompei proposed a new processing method including a double integration and a square root operation [33]. In the work [34], Pompei developed an audible, small deformation, and practical parametric array for the first time. However, the inverse processing method proposed in [34] may not be suitable for practice since the integration shifts most of the signal power to the lower frequency components, and the useful signal generated from the nonlinear acoustic interaction is relatively weak. To resolve this problem, Li proposed some novel preprocessing methods to generate LFM signals, digital modulated signals, and a Ricker wavelet [35]. It is one of the most successful applications of the self-demodulation model to generate a Ricker wavelet in the secondary acoustic field. Due to the fact that a Ricker wavelet can be derived from the second order derivative of Gaussian wave, it is easy to generate a Ricker wavelet in the secondary acoustic field via taking the square root of the Gaussian wave and modulating it onto the carrier. Ricker wavelet can also be generated by simply using half-cosine wave passing through a low pass filter as the envelope of primary wave. In practice, transmitting transducer always has the effects of a band-pass filter, which equals envelop passing through a low-pass filter. Therefore, a half-cosine wave is a better choice due to its simplicity and practicability. Featuring with large bandwidth and good phase characteristics, Ricker wavelet is widely used in seismic exploration, sub-bottom profiling, and the detection of buried targets [36,37,38].

**Figure 5:**
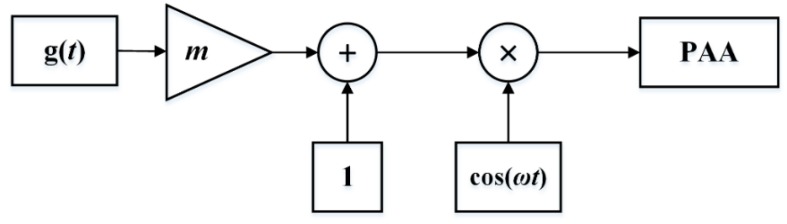
Block diagram of DSBAM.

**Figure 6:**
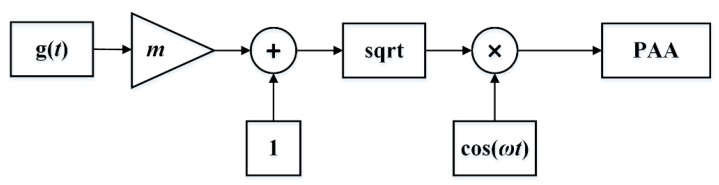
Block diagram of SQRAM.

**Figure 7:**
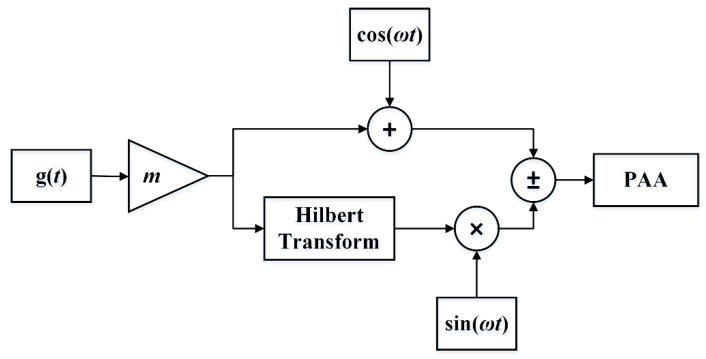
Block diagram of SSBAM.

Pre-processing is essential for generating expected signals, and modulation techniques play an important role in reducing the distortion introduced by the self-demodulation process. There are three common modulation methods, which are double sideband amplitude modulation (DSBAM), square-root amplitude modulation (SQRAM), and single-sideband amplitude modulation (SSBAM). Figure 5, Figure 6 and Figure 7 show the block diagram of the DSBAM, SQRAM, and SSBAM.

An example is given to compare the performance of reducing distortion for different modulation methods. We use a single-frequency sine wave as the modulation signal, and then analyze the spectrum of the self-demodulated signals obtained by these three modulation methods.

For the DSBAM, the modulation envelop is given as E(t)=1+mg(t), where m is the modulation index and g(t)=sin(ωt) is the input signal. Substitution of E(t) into Equation (7) yields:

(10) $$p_{d}=\frac{\beta p_{0}^{2}S_{0}}{8\pi \rho_{0}c_{0}^{4}r\alpha_{0}}(m^{2}\omega^{2}\cos(2\omega t)-m\omega^{2}\sin(\omega t))$$

For the SQRAM, the modulation envelop is given as $E(t)=\sqrt{1+mg(t)}$, where m and g(t) have the same definition as in Equation (10). The difference frequency signal pressure is:

(11) $$p_{d}=-\frac{\beta p_{0}^{2}S_{0}}{16\pi \rho_{0}c_{0}^{4}r\alpha_{0}}m\omega^{2}\sin(\omega t)$$

For the SSBAM, the demodulated signal is:

(12) $$p_{d}=-\frac{\beta p_{0}^{2}S_{0}}{8\pi \rho_{0}c_{0}^{4}r\alpha_{0}}m\omega^{2}\cos(\omega t)$$

From Equations (10)–(12), one derives that if the modulation signal is a single-frequency signal, the distortion of DSBAM is the second harmonic distortion which is in direct proportion to the square of the modulation index, while both SQRAM and SSBAM do not have harmonic distortions. Therefore, DSBAM is not a preferred technique because it incurs high distortion at high m. By reducing the modulation index m, there is a tradeoff between sound pressure level of the demodulated signal and lower distortion, which is not desirable for practical applications. SQRAM is able to reduce distortion, but the transmitting transducer with large bandwidth is required to generate the infinite harmonics introduced by the square root operation [39,40]. Moreover, the amplitude of the demodulated signal generated by SQRAM is half of that generated by SSBAM. In conclusion, SSBAM guarantees the best performance among the three methods, which can reduce the distortion and power consumption simultaneously. Smith gave the conditions for the distortionless transmission of analog modulation schemes using the PAA [41]. He pointed out that SSBAM was able to recover the information efficiently, and it may be sent free of distortion by suitably selecting the carrier frequency, which is consistent with our analysis.

## 4. Design Considerations and Implementation Issues

### 4.1. Design Considerations of PAA Transducer

The secondary source level and the beam-width of the PAA are determined by various factors in a complex manner. Main controlling factors include the primary source level, the step down ratio (i.e., ratio of primary frequency to secondary frequency), the transducer aperture, and the small signal absorption coefficient. Vyas and Raj gave detailed design considerations of the PAA [42]. They pointed out that the design of the PAA involved a compromise between various parameters, such as primary frequencies, secondary frequency, transducer array size and the resulting complexities of the transducer design. The primary frequency governs the virtual array length, cavitation threshold and the transducer size. If high primary frequencies are chosen, the transducer size (for required directivity) may turn out to be small to handle the required powers. If lower primary frequencies are chosen, the corresponding array size may turn out to be too large to carry and install conveniently. Considering the virtual array length, one is tempted to choose high primary frequencies because lower primary frequencies would mean that in any given range, there are only few primary frequency wavelengths available for mixing resulting in lower secondary source levels. However, higher primary frequencies also lead to an increase in step-down ratio, which is associated with lower conversion efficiency and thus lower secondary source level. One way to enhance conversion efficiency and secondary source level is to increase the secondary frequency, but it also increases the absorption attenuation and thus result in poorer penetrability. This is especially problematic in the sub-bottom profile measurement, where low frequency is preferred so as to improve penetration capability. Generally, primary frequencies ranging from 30 to 100 kHz may be a reasonable choice, and a step-down ratio between five and twenty would provide the desired performance. Besides, the selection of the transmitting transducer also has a great impact on the performance of the PAA. On the one hand, the transducer array should have a large surface area to ensure high transmitted power and high source levels. On the other hand, the law of Rayleigh limitation demonstrates that a large aperture of transducer is needed for a low-frequency sonar in order to get a fine directivity, while transducers operated at high frequencies usually do not need very large aperture to gain a sufficient directivity. In general, the array size is controlled by the choice of primary frequency, and the care should be taken that for a particular array size, the maximum transmittable powers are achieved without reaching the cavitation threshold.

Kopp proposed some design equations to optimize the parameters of the PAA from an engineering viewpoint [6]. The design principles proposed by Kopp are based on the values of three distances mentioned in Section 2: the efficient length of the PAA $R_{A}$, the Rayleigh distance $R_{F}$, and the shock distance $R_{s}$. The optimal design is to make these three values equal (i.e., $R_{F}=R_{A}=R_{s}$). $R_{A}=R_{F}$ implies that the array diameter is matched to the frequency, and $R_{A}=R_{s}$ indicates that acoustic power is not wasted through excess attenuation. Given a specific array size, we can easily obtain the optimum primary frequency and transmitted power using the above equations. In the practical applications of underwater acoustic engineering, however, it is difficult to fulfill such ideal condition. Specifically, the absorption coefficient α is small underwater, so the efficient length of the array $R_{A}$ is large. However, the Rayleigh distance $R_{F}$ cannot be too large since the signal used in underwater acoustic engineering usually has relative low frequency and the transducer size is limited. As a result, it is almost impossible to realize $R_{A}=R_{F}$ in practice. The design of a PAA depends upon the type of application and the mathematical model of the secondary sound field. Each design of PAA is unique and requires specific analysis.

### 4.2. Implementation Issues

The PAA system consists of three main modules, namely, signal processing, power amplifier and transmitter (shown in Figure 4). In addition to the design of the PAA transducer, the implementation of signal processing and amplifier is also important for developing a PAA system. Compared to analog circuit system, a digital signal processor has the ability to process more complicated operations without extra cost, size, and power consumption. As a result, most PAA systems use digital circuits to perform the signal processing block. A field programmable gate array (FPGA) is one of the attractive digital signal processing platforms to implement the PAA because of its flexible configuration and high performance. A single chip microcomputer, as a substitute for FPGA, also can perform signal processing.

To increase transmitting power and improve penetration, the signal must be amplified before being fed into the transducer. The Class-D power amplifier is widely used in the parametric loudspeaker due to its high efficiency and small size. However, Class-D power amplifiers are commonly used to amplify audio signals whose frequency is between 20 Hz and 20 kHz. When the PAA system is applied in underwater acoustic engineering and the signal frequency is higher, other amplifier configurations with broader bandwidth should be used. Svilainis suggested a power amplifier configuration and obtained 50 kHz to 3 MHz bandwidth [43]. The total harmonic distortion of 4% using 3 kΩ load and 400 Vp-p 1 MHz frequency signal was achieved, which indicated the ability to use such a power amplifier for high power and high frequency waveform excitation of transducers. Other researchers have developed power amplifiers for transmitting transducers by various techniques, such as digital predistortion and dynamic current biasing techniques to ensure high-voltage and low-distortion [44], power MOSFET linearizer scheme to improve the gain deviation characteristics of the power amplifier at higher input powers [45], etc.

**Figure 8:**
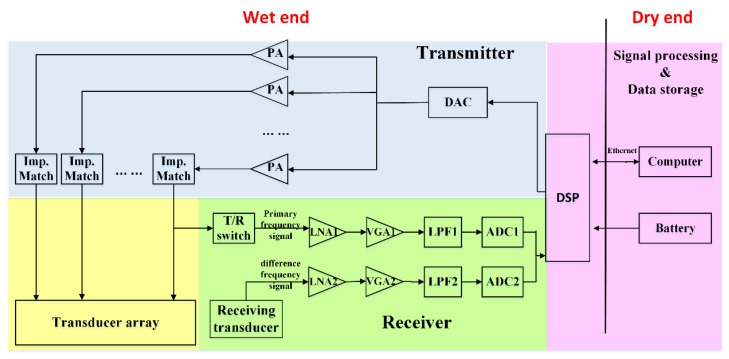
System configuration of a parametric array sub-bottom profiler.

In addition to the signal transmitting circuit, a complete PAA system in practice also requires a signal receiving circuit. As an example, Figure 8 shows the system configuration of a parametric array sub-bottom profiler. The system consists of a dry end and a wet end. The dry end includes a computer serving as a control and display center, and a battery serving as power supply. The wet end is composed of an embedded signal processor (DSP), a transmitting circuit, a primary frequency signal receiving circuit, a difference frequency signal receiving circuit, and a transducer array. The DSP generates the desired signal after receiving the instructions of the upper computer, and then the signal is sent out through the transmitting circuit, which comprises a digital-to-analog converter (DAC), some power amplifiers (PAs) and impedance matching networks (the number of which is in accordance with the transducer elements). In addition to the need for a high excitation voltage and satisfactory transducer elements, the successful transducer excitation also requires impedance matching between the transducer and the excitation source. In most cases, the equivalent impedance of piezoelectric transducers is capacitive. The parasitic input capacitance of the transducer clamps the PA output, and thus reduces the amplifier efficiency. If no electrical impedance matching network (EIMN) is adopted, it will not only seriously affect the power transfer efficiency, but also cause severe heating of the PA, resulting in permanent damage to components. There are many publications on EIMN design for piezoelectric transducers. The main design methods include analytical methods [46,47] and computer aided design (CAD) methods [48,49]. The analytical method is very complicated and requires the analytical form of the transducer, limiting its practice in engineering. The result of the CAD method is usually dependent on the choice of the matching network topology. A relatively simple and effective method is based on the Smith chart, assuming that the bandwidth has an inverse relationship with the quality factor *Q* [50]. Moreover, some researchers use optimization algorithms such as the genetic algorithm to search for optimal EIMN designs [51].

The receiver is divided into the primary and difference frequency receiving circuits. The receiving circuit of the primary frequency signal has sounding function, while the difference frequency signal receiving circuit can provide accurate information of the deeper stratum due to the high directivity and strong penetrability of the difference frequency signal. In addition, the primary receiving circuit provides an important reference for the starting point of the time-varying gain (TVG) compensation to the difference frequency channel. Both primary and difference frequency receiving circuits consist of a low noise pre-amplifier (LNA), a variable gain amplifier (VGA), a low-pass filter (LPF) and an analog-to-digital converter (ADC). The difference between the two receiving circuits is that the primary frequency signal receiver has a transmit/receive (T/R) switch to block high-voltage transmission signals and protect the receiving circuit. In addition, the cut off frequency of the LPF in the difference receiving circuit is lower to ensure to filter out the high-frequency primary signal.

## 5. Applications of the PAA in Underwater Acoustic Engineering

Based on the PAA system, a broadband beam with low frequency, high directivity, and almost without side-lobes can be realized with small-size transducers. PAA is very suitable for high-resolution detection of seabed stratigraphic profiles and represents an important development direction in sub-bottom profile measurement. In addition, the PAA also has many other applications in underwater acoustic engineering, such as underwater acoustic communication, detection of buried targets, doppler sonar log, etc.

### 5.1. Parametric Array Sub-Bottom Profiler

The most mature application of the PAA is the sub-bottom profiler [52,53,54]. Chirp sub-bottom profiler is widely used in the field of traditional acoustic detection [55,56], because Chirp sonar transmits LFM signals with broad bandwidth which carry a lot of information about the submarine stratum. However, its transducer is usually very large and heavy due to the need to generate a low frequency signal with sufficient penetrating power, so the installation is quite inconvenient. In addition, the beam angle of low frequency chirp sonar is large, which results in poor resolution. In contrast, the parametric array sub-bottom profiler has the advantages of low frequency, high directivity, and small size, and thus, it is gaining more and more attention. As an example, Table 1 gives a comparison of the technical specifications of a parametric array sub-bottom profiler (SES-2000 Medium) and a Chirp sub-bottom profiler (BATHY2010).

INNOMAR company in Germany has applied the research results of Underwater Acoustics Research Group of Rostock University to produce a series of SES-96 and SES-2000 parametric array sub-bottom profilers. This product line covers all ranges of water depth from less than one meter to full ocean depth. Applications include geophysical imaging of sediments and sub-seabed structures for dredging purposes, route and offshore site surveys and mapping buried pipelines/cables. For instance, SES-2000 deep-36 Sub-Bottom Profiler can work in up to 6000 m under water. Its primary frequency is 36 kHz, and the frequency band of the secondary frequency is 1–10 kHz. In addition, its source level reaches up to 246 dB re 1μPa at 1 m, so the sediment penetration is up to 150 m.

Another advanced parametric sonar system, PARASOUND sub-bottom profiler produced by ATLAS company, is widely used in marine surveys [57,58]. PARASOUND P70 is suitable for underwater operations from 10 m to 10000 m. It operates at primary frequencies of 18–39 kHz to provide secondary frequencies as low as 500 Hz. With a secondary parametric source level of approximately 206 dB, it provides an ability of bottom penetration more than 200 m, with a high resolution of less than 15 cm depending on bottom characteristics. Moreover, the system benefits also include heave, roll, pitch compensation for beam stabilization and electronic beam steering for larger detection coverage.

TOPAS PS18 and PS40 sub-bottom profilers are produced by Kongsberg company. The TOPAS system can operate with various signal waveforms to achieve optimum performance: Ricker pulses are used for very high resolution work, Chirp pulses are used for deep water, high penetration work, and CW pulses are used for narrow band, frequency sensitive work. The transmitted acoustic beam is electronically stabilized in both roll and heave ensuring that the insonified area on the sea floor is accurately positioned. In addition, the transmitter can be used in a sequentially beam steering mode for covering a larger sector. Due to these advantages, TOPAS has been widely used in marine surveys, e.g., detecting Atlantic herring [59], investigating cold-water coral structures [60], analyzing the structure of sediments in the Scotia Sea [61], etc.

### 5.2. Parametric Underwater Communications

High performance underwater acoustic communications require low carrier frequencies for low channel attenuation, high bandwidths for high data rates and narrow beam for less multipath effect. These various and contradictory requirements can be satisfied by employing PAA. MPSK, MFSK, OFDM, and many modulation methods combined with the nonlinear effect of the PAA, are applied to underwater communications and achieve good performance [62,63,64,65]. Parametric sonar with M-ary DPSK modulation was used for underwater digital communication in [63]. The system can realize real time acoustic communications at ranges of tens of kilometers and can achieve data rates of 1, 2, and 3 kbit/s for 2-, 4-, and 8-DPSK, respectively. Further, the narrow beam width achieved by the PAA helps to secure the data from a spatial point of view, thus the PAA shows the application potential in covert underwater acoustic communication [65]. This is especially useful in military applications where the secrecy of transmission signal is extremely important. PAA is also used in under-ice environment, a direct sequence spread spectrum system based on the PAA was proposed and verified by Tang [66].

### 5.3. Detection of Buried Targets

Efficient and accurate detection of objects buried in the seafloor is a major requisite for both of the military and the public. PAA is the first choice for the detection of buried targets [37,67,68]. However, most existing parametric sonars operate in single-beam modes, and the detection efficiency is therefore relatively low, which fails to meet the military requirements. So, the multi-beam parametric array is a hot research topic for scholars all over the world [52,69,70]. For example, a parametric array whose acoustic beam can be steered electronically was proposed by Dybedal and Boe [71], its sequential scanning mode of operation enables it to cover a 3D volume of sub-bottom sediments. Until now, however, there have been no multi-beam parametric arrays that are matured in operation. Deploying PAA onto unmanned underwater vehicles (UUV), autonomous underwater vehicles (AUV) and remote operated vehicles (ROV) offers advantages of low cost, reduced operator risk and potentially improved coverage rates. Therefore, the combination of the PAA and underwater vehicles shows great potential for development in the domain of buried objects detection [72,73].

### 5.4. Long-Range Ocean Research

Long range acoustic propagation in the ocean is characterized by strong mode coupling. Resolving the travel time variability in several tenths of milliseconds for multi-paths in the ocean waveguide usually requires sophisticated signal processing techniques or single mode excitation. The directivity pattern of the PAA can be very sharp and almost independent of the wave frequency. Therefore, PAA can provide the broad frequency band, single mode acoustic source needed for propagation in shallow water waveguides [74]. A long-range ocean experiment using a PAA for up to 1000 km range signal propagation was performed in the early 1990 s [75,76], which has proven the technical advantages of the PAA that make it “a perfect tool for ocean acoustics”.

## 6. Technical Challenges of the PAA

### 6.1. Improvement of Conversion Efficiency

PAA provides a number of advantages over its linear counterpart, but it also has some disadvantages such as low conversion efficiency and some complexity in design. The typical conversion efficiency of the PAA is nearly 1%, greatly hindering the further development and application of the PAA. Improving the conversion efficiency is thus an urgent task. From Equations (1) and (2), one derives that increasing the primary source level and secondary frequency well contributes to improving the conversion efficiency. However, the primary source level cannot be increased indefinitely due to the phenomenon of acoustic saturation. In addition, the maximum transmittable power should be below the cavitation threshold. Likewise, the increase of secondary frequency is limited as well, because higher secondary frequency coupled with higher absorption attenuation leads to lower penetration depth or range. To enhance the conversion efficiency, a number of recent studies investigate the optimization of the transducer material and design [77,78,79]. Some researchers fabricated capacitive micromachined ultrasonic transducers (CMUTs) with vacuum-sealed cavities and used them to project directional sound using PAA. The devices were used to produce a narrow (8.7°) beam of 5 kHz sound, which at 3 m was 58 dB [77,78]. A more recent work on the design of the parametric array transducer was presented by Ahn, who integrated a dual-resonant-frequency PZT rods into a thinner polymer plate to increase the radiation surface and thus to enhance power efficiency [79]. The difference frequency wave generated by the PAA had sound pressure levels of 150 dB re 1 μP (@ 30 kHz) and a high directivity of 3.4° half-power beam width.

### 6.2. Realization of Multi-Beam Detection

The receiving mode of the PAA is generally low-frequency, wide-beam receiving. If the low-frequency narrow-beam receiving mode is adopted, the aperture of the receiving array will inevitably become larger, which will lose the main advantage of the parametric array with small aperture of the transducer. Therefore, the PAA does not have the ability of a conventional multi-beam sonar to distinguish signals in different directions by receiving multiple beams. The lower detection efficiency of the single-beam operation mode is a limiting factor for the wider use of the PAA. Therefore, how to improve detection efficiency of the PAA is gaining more and more research attention.

The phased parametric array is one of the most promising ways to achieve multi-beam scanning. However, during the beam steering process, the difference frequency wave of phased parametric array may have problems of broadening beam lobe and generating high intensity grating lobe and side-lobe. There are a few pioneer researches on the beamforming method for phased parametric array [52,69,80,81,82], but this technique is not matured enough to be of practice use. For a digital beamsteering system, the available steerable angle is often restricted by the sampling interval of the digital system. As the sampling frequency is not high enough, the smallest steering angle available is relatively large, making it difficult to distinguish signals in different directions. A number of fractional delay or frequency domain algorithms have been developed to improve the steering angle, but some of the algorithms requires high computational load and others introduce errors during the process. Gan proposed a digital beamsteerer for difference frequency in parametric array, which is able to steer to small angles without the need to increase the DSP board sampling frequency or implement fractional delay [80]. However, this method requires multiplications and additions for each sampling period, which to some extent increases computational complexity.

Frequency division multiple access (FDMA) and code division multiple access (CDMA) technologies can distinguish beams in different directions from the perspective of signal spatial characteristics, thus realizing multi-beam detection through the PAA. However, considering the self-demodulation effects of the PAA and the requirements for signal bandwidth, pulse length, and reverberation suppression, the practical application effects of these methods are limited in complex marine environments. In conclusion, there is a need for further research to realize effective multi-beam detection by the PAA.

## 7. Conclusions

This review paper gives an overview for a wide range of important works in the field of the PAA. Based on nonlinear acoustics, the theory of the PAA has made considerable progress in recent decades. We examine some of the important advances in the PAA, including theoretical developments in nonlinear acoustics, mathematical modeling for the PAA, signal processing techniques, and design guidelines of the PAA for optimum performance. With the development of relevant theories and technologies, PAA has moved from theory to implementation and application. Due to its outstanding advantages, PAA is widely used in the air and underwater, and this paper focuses on its applications in underwater acoustic engineering. Despite wide applications of the PAA, there are still some technical challenges to overcome. In particular, we highlight the technical challenges concerning conversion efficiency and detection efficiency. Compared with the traditional linear sonar system, the secondary source level of the PAA is much lower due to the low conversion efficiency. Increasing the secondary frequency is one of the feasible methods to enhance the secondary source level and more and more PAA systems with relatively high secondary frequencies and small step down ratios are being applied in underwater acoustic engineering. Further, improved transducer performance also improves conversion efficiency and secondary source level. Some new materials and designs of transmitting transducers are proposed for the PAA. Another focus of future research will be to develop the multi-beam parametric array and improve the detection efficiency. To realize effective multi-beam transmission by the PAA, we should aim to develop the digital beamsteerers with small steering angles and improve the beamforming methods to suppress side lobes. We believe the improvements of conversion efficiency and detection efficiency will further promote the application of the PAA.

## Figures and Tables

**Table 1:** Comparison of technical specifications of sub-bottom profilers.

Technical Specifications	SES-2000 Medium Sub-Bottom Profiler	BATHY 2010 Sub-Bottom Profiler
Water Depth Range	2–2000 m	10–12000 m
Sediment Penetration	70 m (depending on sediment type and noise)	200 m (depending on sediment type and noise)
Range Resolution	1 cm	8 cm
Beam Width (−3dB)	1.0° × 1.0°	−3dB:31°, −6dB:45°
Primary frequency (PF)	100 kHz (frequency band 85–115 kHz)	3.5 kHz (for sediment penetration) 12 kHz (for sounding)
PF Source Level	>247 dB re 1 μPa at 1 m	>156 dB re 1 μPa at 1 m
Acoustic Power	5.5 kW	4 kW
Secondary frequency	Selectable: 3.5, 5, 6, 8, 10, 12, 15 kHz	——
Pulse Width	0.07–3.5 ms	0.2–50 ms
Pulse Type	Ricker, CW, LFM chirp	CW, LFM chirp
Transducer Size	W 0.50 m × D 0.50 m × H 0.12 m	W 0.483 m × D 0.635 m × H 0.40 m
